# Frequent and genetically diverse *Plasmodium vivax* relapses contribute to malaria transmission in Cambodia

**DOI:** 10.21203/rs.3.rs-9199901/v1

**Published:** 2026-04-02

**Authors:** Dynang Seng, Katie Ko, Agnes Orban, Sokleap Heng, Malen Ea, Baura Tat, Lionel Brice Feufack Donfack, Virak Eng, Kieran Tebben, Nimol Khim, Thierry Lefèvre, Anna Cohuet, Cecile Sommen, Claude Flamand, David Serre, Jean Popovici

**Affiliations:** 1. Malaria Research Unit, Institut Pasteur du Cambodge, Phnom Penh, Cambodia; 2. Institute for Genome Sciences, University of Maryland School of Medicine, Baltimore, USA; 3. MIVEGEC, IRD, CNRS, University of Montpellier, 34090 Montpellier cedex 5, France; 4. Epidemiology and Public Health Unit, Institut Pasteur du Cambodge, Phnom Penh, Cambodia; 5. Infectious Disease Epidemiology and Analytics, Institut Pasteur, Paris, France

**Keywords:** *Plasmodium vivax*, malaria, relapses, hypnozoites, transmission, complexity of infection

## Abstract

*A* unique feature of *Plasmodium vivax* is the presence of dormant hypnozoites that cause relapses weeks after the initial infection. While relapses may account for the majority of vivax malaria cases, the determinants of relapse periodicity, the genetic relationships between parasites, and the transmission potential of relapsing parasites in endemic populations remain poorly understood.

We characterized *P. vivax* relapses in a cohort of 59 Cambodian patients treated with artesunate and relocated to a malaria-free area to prevent reinfection. Participants were followed every two days for 90 days using qPCR and microscopy. Genomes of blood stage parasites were sequenced to determine clonality and genetic relatedness between initial and relapsing infections. Membrane-feeding assays using *Anopheles dirus* mosquitoes were performed to evaluate the transmission potential of relapse infections.

Relapses occurred frequently, with 81% of the patients experiencing at least one relapse within 90 days, with up to four consecutive relapses per individual and a median time to first relapse of 20 days. Genome sequencing revealed that relapsing parasites in monoclonal infections could be genetically identical, siblings of, or unrelated to the parasites present at enrollment. Patients with polyclonal infections at enrollment experienced significantly more relapses than those with monoclonal infections, suggesting that infection complexity may reflect the heterogeneity of the hypnozoite reservoir. Parasite growth rates during relapses varied widely among individuals but were associated with the degree of relatedness to those present in the initial infection. Despite low parasitemia and the absence of symptoms in most cases, relapse infections were frequently infectious to mosquitoes in membrane-feeding assays.

Our findings show that *P. vivax* relapses occur commonly and frequently, and often involve genetically distinct parasites, reflecting complex hypnozoite reservoirs. Importantly, even low-density relapse infections can transmit efficiently to mosquitoes, highlighting the critical role of hypnozoites in sustaining malaria transmission and the importance of radical cure strategies for vivax malaria elimination.

## Introduction

Malaria poses a significant burden on global health, with an estimated 282 million cases and 610,000 deaths reported in 2024^1^. The disease is caused by parasites of the genus *Plasmodium*, with six species infecting humans. *P. falciparum* is the predominant species in Africa, while *P. vivax* is responsible for the majority of cases outside Africa, with 2.5 billion people at risk of infection^2^. *P. vivax* shows distinct biological features compared to *P. falciparum* and notably can produce a unique dormant liver stage known as hypnozoites. Hypnozoites can remain quiescent for weeks, months, or even years before unknown mechanisms trigger their activation and subsequent schizogony, resulting in relapsed blood-stage infections. These hypnozoites constitute a significant challenge for malaria control and elimination strategies: in some endemic areas, 80–90% of *P. vivax* infections are estimated to result from relapses rather than newly acquired infections^3^. In addition, the only drugs available to target hypnozoites are primaquine and tafenoquine, which can have major side effects in patients with glucose-6-phosphate dehydrogenase deficiency (G6PDd), complicating their use in endemic areas. Another biological characteristic of *P. vivax* that complicates vivax malaria control is its ability to produce gametocytes very early during the blood stage infection. Contrary to *P. falciparum*, *P. vivax* can be transmitted to mosquitoes before the onset of symptoms, and therefore before a patient seeks treatment^4,5^. Individuals living in endemic areas can acquire partial immunity through repeated inoculations of *P. vivax* and control their infection (i.e., they are often infected but asymptomatic). However, several studies have shown that their contribution to the disease transmission is not negligible, although the exact extent to which asymptomatic individuals contribute to transmission in endemic areas remains debated^6–8^. A recent study employed serial dilution of gametocytes to assess transmission rates to mosquitoes and showed that typical parasitemia levels in asymptomatic carriers could contribute significantly to transmission potential^9^. However, there are still significant knowledge gaps in our understanding of *P. vivax* transmission at low parasitemia and, in particular, the transmission potential of relapsed parasites to mosquitoes remains undocumented.

In general, we know very little about relapses or the biology of hypnozoites. The vast majority of previous studies examining relapses relied on experimental infections of neurosyphilis patients^10,11^ or returning travelers (or soldiers) from malaria-endemic areas^12^. These early studies have shown that relapse periodicity was driven by several factors, such as the geographic origin of *P. vivax* (i.e., 3 to 4 weeks between relapses in tropical regions vs. several months for parasites from temperate areas) or the sporozoite inoculum (the bigger the inoculum, the shorter the interval)^12^. However, since these studies were conducted mostly in non-immune individuals with little or no prior exposure to malaria, they may not accurately recapitulate the patterns of relapses for people living in endemic regions, where constant exposure to parasites and environmental and genetic factors may influence relapse patterns differently. Studying relapses in naturally infected individuals living in an endemic area poses inherent challenges, such as the difficulty of distinguishing reinfections from relapses or recrudescence^13–15^, or the duration of post-treatment prophylaxis that could mask early relapses, and depends on the half-life of the antimalarial drug used^16^.

Here, we analyzed patients enrolled in a clinical trial designed to evaluate primaquine efficacy^17^. Specifically, we analyzed relapses among patients enrolled in the comparator arm who were not treated with primaquine at enrollment. The use of artesunate, a short-lived blood-stage antimalarial to clear the initial blood stage infection (resulting in minimal post-treatment prophylaxis) combined with the relocation of the patients into a transmission-free area during the follow-up (to prevent re-infections), allowed rigorous characterization of relapses *in vivo* and enabled us (i) to investigate the dynamics of, and genetic relationship among relapses, (ii) to determine the impact of relapses and host factors on the growth rate of *P. vivax* parasites upon relapses, and (iii) to evaluate the transmission potential of relapsing parasites.

## Materials and Methods

### Study design, patient enrollment, and follow-up

We analyzed data obtained from patients enrolled in a no-primaquine arm of a clinical trial to determine the therapeutic efficacy of primaquine. A total of 61 patients with *P. vivax* mono-infection were recruited in this arm, but 2 patients withdrew shortly after enrollment and are not included in downstream analyses. Details about the recruitment of patients and the clinical trial were reported elsewhere^17^. All patients received a supervised standard 7-day course of artesunate monotherapy (2mg/kg/day) (RT Nate, Bonn Schtering Bio Sciences, India). Upon inclusion, participants were relocated to a non-transmission area (Aoral town, Western Cambodia) to prevent reinfections from mosquito bites. At enrollment, medical history was recorded, and physical, clinical, and biological examinations were performed. After 7 days of artesunate treatment and daily follow-up, patients were followed every second day for 90 days with capillary blood collection, clinical examination, and axillary temperature measurement at every time point. After initial parasite clearance, patients with a *P. vivax* recurrence detected by PCR and confirmed by microscopy were retreated with the same 7-day regimen of artesunate, and monitoring continued until the end of the study period. Before each treatment, including after enrollment at the beginning of the study, a hematological test complete blood count was performed. Methaemoglobin percentage (SpMet), oxygen saturation (SpO_2_), carboxyhemoglobin (SpCO), pulse rate, and perfusion index were measured noninvasively using a RAD-57 pulse CO-oximeter (Masimo, CA, USA). G6PD-normal patients were provided with primaquine at the end of the 90-day follow-up period for self-administration. Note that for some patients, relapses were detected by PCR and did not become microscopy-positive by day 90, at which point patients were referred to a local health facility for standard anti-malarial treatment (artesunate-mefloquine) without blood collection for genomic and clinical analyses. For a subset of patients and after amendment to the study protocol, blood samples were also collected for mosquito membrane-feeding assays at enrollment and upon confirmed *P. vivax* recurrence.

### Microscopy, real-time PCR, and determination of *P. vivax* growth rates

Using Giemsa-stained thick films, parasitemia was estimated by counting the number of parasites per 200 white blood cells (assuming a white blood cell count of 8,000/μL). DNA was extracted from 50 μL of each blood sample using a QIAamp DNA Blood Mini Kit (Qiagen, Courtaboeuf, France), and the presence and species of *Plasmodium* parasites were determined by RT-PCR^18^. For sub-microscopic infections upon relapse, parasite density was estimated by quantitative PCR (qPCR) of *P. vivax* cytochrome B, normalized to human β-tubulin DNA^18,19^. Each PCR amplification was performed in a 20 μL reaction containing 0.25 μM primers, 1X Evagreen SYBR mix, and 1 μL of DNA. All reactions were conducted under the same conditions: 95 °C for 15 minutes, followed by 45 cycles of 95 °C for 15 seconds, 60 °C for 20 seconds, and 72 °C for 20 seconds, followed by melting curve analysis. The growth rate of *P. vivax* upon relapses was expressed as the slope of the linear regression of log-transformed parasite densities.

### Experimental mosquito infections

Membrane-feeding assays (MFAs) were conducted using laboratory-reared *Anopheles dirus*, a primary vector in Southeast Asia, to assess the transmission potential of *P. vivax* relapses. In brief, blood samples were collected at enrollment (D0) and upon relapses (within 24h to 48h of microscopy detection and prior to re-treatment) and used for membrane feeding. Patient serum was removed and replaced with the same volume of naïve human AB serum to 50 % hematocrit. Note that for some experiments, serum was not replaced, and whole blood was used directly. The mixture was then fed for 1h to 5–7-day-old female mosquitoes (n=80 per feed) via an artificial membrane attached to a water-jacketed mini-feeders maintained at 37 °C using a pump and water bath^20^. Unfed and partially fed females were discarded, while fed *A. dirus* mosquitoes were maintained at 26 °C and 80% humidity and fed a 10% sucrose solution containing 0.05% PABA dissolved in distilled water. On the sixth day post-blood meal (dpbm), 20 mosquitoes were dissected under serum-replacement conditions, and 50 under no-serum-replacement conditions.

The oocyst prevalence (the proportion of blood-fed mosquitoes with at least one oocyst in the midgut) and oocyst intensity (oocyst count per infected mosquito) were recorded.

### Whole genome sequencing and determination of the complexity of infection

We extracted parasite DNA from leukocyte-depleted blood samples using the DNeasy blood and tissue kit (Qiagen) and prepared whole genome sequencing libraries using the NEBNext^R^ Ultra^™^ II FS DNA Library Prep Kit for Illumina NovaSeq 6000 to generate 25–50 million paired-end reads of 100 bp per sample. We used Hisat2^21^ with default parameters to map the reads to the P01 reference genome^22^ (version 67). Samples with an average coverage of less than 50X were not further analyzed. We then estimated whether each sample was monoclonal or polyclonal by using GATK^23^ to call nucleotide variants in nontelemetric regions and excluding multigene families (PIR genes, VIR genes, merozoite surface protein genes, and tryptophan-rich protein genes). We considered only positions with at least 20X coverage in at least 80% of the samples and only polymorphic positions with two alleles. We then used the R package moimix^24^ to estimate polyclonality: infections with a F_ws_ ≥ 0.95 were considered as monoclonal, and those with F_ws_ < 0.95 were considered as polyclonal. We analyzed the genetic relatedness among monoclonal samples by estimating Identity by Descent (IBD). For the IBD analysis, we again used the same filtered nucleotide variants as for estimating polyclonality (see above), and analyzed positions that were shared in at least 80% of samples with hmmibd-rs^25^. We then used the fraction of sequence IBD between each pair of samples to generate a distance matrix and a Neighbor-Joining tree in MEGA11^26^.

### Statistical analyses

All statistical analyses were performed using R (version 4.5.1) and R Studio (2025.05.1). The Kruskal-Wallis or Mann-Whitney test was used to compare parasitemia between infection episodes (D0: at enrollment, R1: 1^st^ relapse, R2: 2^nd^ relapse, R3: 3^rd^ relapse), growth rates, followed by Dunn’s post hoc test for multiple pairwise comparisons. Fisher’s exact test was used to compare proportional data. The effect of clinical parameters on the number of relapses was assessed using multivariable Poisson regression within a generalized linear model (GLM) in the stats package, or multivariable logistic regression to assess the effect of clinical parameters on the risk of relapse. To assess the effect of the relapse ranks on hematologic parameters, a linear mixed-effects model (LMM; lme4 package) was applied, with the relapse ranks as a fixed effect and participant ID as a random intercept. The effect of relapse ranks and clinical parameters on the parasite growth rate were analyzed using a linear mixed-effects model (LMM; lme4 package), with participant ID as a random effect. The effects of log-transformed parasitemia, infection episodes, age, and sex of the patient on oocyst prevalence and oocyst intensity were analyzed using a hurdle model. The model included (i) a binomial mixed-effects component to assess the probability of observing at least one oocyst (oocyst prevalence) and (ii) a zero-truncated negative binomial mixed-effects component to model oocyst count (oocyst intensity). The effects of log-transformed parasitemia and the complexity of infection on oocyst prevalence and intensity were also analyzed. Both components were fitted using the glmmTMB package, with participant ID included as a random intercept. Model assumptions were evaluated using the DHARMa package. The overall fixed effects from Poisson and Logistic regression were assessed using the Wald chi-square test via the Anova function in the “car” package and using Type II ANOVA for linear models. For a detailed assessment of specific individual factor levels and continuous predictors, individual coefficient z-tests were obtained from the “summary”, representing comparisons against the reference level. The contribution of random intercept in LMMs was evaluated using the ranova function from the “lmerTest” package. All reported p-values are two-sided and were considered statistically significant at *p* < 0.05.

### Ethical statement.

The protocol was approved by the National Ethics Committee for Health Research of Cambodia (158-NECHR) and by the University of Maryland IRB (HP-00091095). The study was overseen by the NIH DMID (Protocol 20–0010). All patients or their guardians provided written informed consent, and written assent was obtained for all patients aged 15–18 years old.

## Results

### Relapses occur frequently in relocated patients after artesunate treatment

**Fig. 1.**
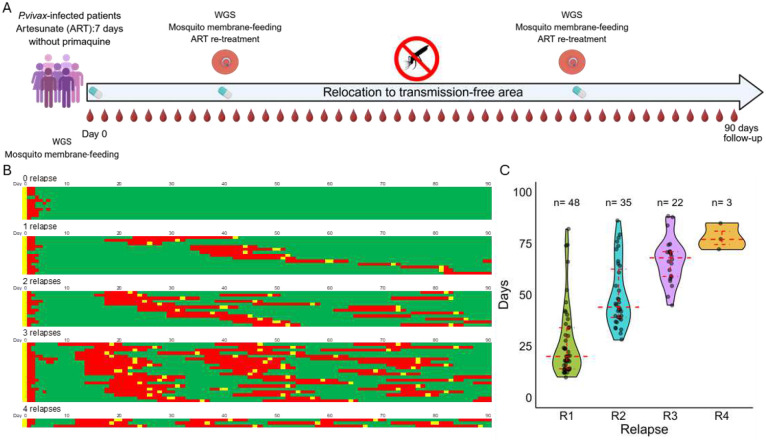
*P. vivax* relapses are common and frequent after treatment of blood stage infections. **A**. Overview of the study design. Patients with *P. vivax* mono-infections were treated with 7 days of artesunate, relocated to a malaria-transmission-free town, and followed for 90 days. Capillary blood samples were collected every second day for qPCR-based detection of parasites. As soon as a *P. vivax* recurrence was confirmed by microscopy, patients were treated again with the same supervised 7-day artesunate regimen, and follow-up continued until day 90. Upon recurrence, venous blood was collected before treatment for genomic analyses (WGS: whole-genome sequencing), and, for a subset of patients, for experimental infections of *Anopheles dirus* mosquitoes. **B.** Representation of relapses detection and treatment among 59 patients followed for 90 days. Each row represents one individual patient. Green: *P. vivax*-negative PCR; Red: *P. vivax*-positive PCR; yellow: artesunate treatment initiation. **C**. Day of first PCR detection of first, second, third, and fourth relapses. The x-axis shows the different relapse ranks, while the y-axis shows the days on which the relapse was first PCR-detected. The red dashed crossbars indicate the median, and error bars represent the interquartile range. R1, R2, R3, and R4 denote first, second, third, and fourth relapses, respectively.

59 patients, enrolled in the comparator arm of a primaquine efficacy clinical trial (see Materials and Methods^17^), were treated with a supervised 7-day course of artesunate monotherapy (ART, 2mg/kg/day). Upon enrollment, patients were relocated to an area with no malaria transmission and monitored every second day for 90 days (Fig. 1A). All infections were cleared of microscopically detectable parasites within 3 days of initiation of artesunate treatment and were PCR-negative by day 6 (Supp. Table 1).

Among the 59 patients, 48 (81%) experienced at least one recurrence of *P. vivax* parasites within 90 days, with an average number of 2.25 recurrences (range 1–4) and among those, the majority had three recurrences (Fig.1B, Supp. Fig. 1). All recurrences were also efficiently cleared by artesunate and no microscopically detectable parasites remained after 3 days. The absence of changes in clearance rates following repeated treatment supports the hypothesis that the observed recurrences are unlikely to be recrudescence of resistant or persistent parasites (i.e., treatment failure). As reinfections were prevented by relocating participants to a transmission-free town, we ascertained that these parasite recurrences were relapses from hypnozoite reactivation (but see also discussion below). In addition, patients enrolled in the same study and treated with the same dose of artesunate, but who additionally received a high dose of primaquine, remained free of parasites for at least 60 days, further supporting the hypothesis that the recurring parasites derive from relapses.

The earliest relapse was detected by PCR 10 days after enrollment, while the latest first relapse observed during this follow-up period occurred on day 82, with a median of 20 days (IQR:14–34) from enrollment to the first relapse (Fig. 1C). Note also that 11 patients did not relapse during the monitoring period.

### Clinical parameters at enrollment and dynamics throughout *P. vivax* relapses

To assess whether any clinical parameter was associated with the total frequency of *P. vivax* relapses, we analyzed clinical data collected at enrollment, prior to treatment initiation, and tested associations between these clinical parameters and the total number of relapses over 90 days. None of the parameters were significantly associated with the number of relapses (Supp. Table 2), nor with the patient having a relapse or not (Supp. Table 3), with the possible exception of neutrophil counts, which showed a borderline positive association with the total number of relapses (LRT $\chi_{1}^{2}=3.82$, p = 0.05), warranting further investigation.

At enrollment, all patients presented with uncomplicated malaria symptoms, including fever, chills, and headaches. Some patients also reported additional symptoms, including dizziness, vomiting, nausea, or abdominal pain. Upon relapses, patients were re-treated with artesunate within 24h to 48h of collection of the capillary blood sample from which parasites were identified by microscopy, regardless of whether they were symptomatic. Therefore, parasite densities of relapses were lower than parasitemia at enrollment (Kruskal-Wallis, p < 0.0001, but there was no difference in parasitemia between relapses, p = 0.98, Supp. Fig. 2). Correspondingly, most relapses were asymptomatic:13% (6/47) of patients experienced symptoms at the first relapse, 18% (6/33) at the second, and 22% (4/18) at the third (Supp. Table 4), and there was no significant difference in the presence of symptoms across relapses (Fisher’s exact test, *p* = 0.732).

At enrollment, the majority of patients exhibited systemic signs of infection, with 97% (57/59) demonstrating thrombocytopenia (platelet count < 200 × 10^9^/L). Hemoglobin-related parameters were also markedly abnormal: 66 % (39/59) had a low mean corpuscular hemoglobin (MCH < 27 pg), 58% (34/59) had low mean corpuscular volume (MCV < 80 fL), and 41% (24/59) had hemoglobin levels below 13 g/dL. Upon subsequent relapses after initial parasite clearance, the proportion of individuals with abnormal values decreased across most parameters. Hematological recovery was evident, as abnormal RBC counts, hemoglobin level, and hematocrit value became less frequent with each relapse. However, eosinophilia exhibited an opposite trend. While relatively infrequent at enrollment, 14% (8/59), the frequency of patients with eosinophilia (> 0.5 g/L) increased during relapses and was observed in 47% (21/45) patients during the first, 48.5% (16 /33) during the second, and 61% (11/18) during the third relapse (Supp. Table 4).

We then tested whether the relapse ranks affected clinical parameters. When analyzing all relapses, seven parameters (red blood cell counts, hemoglobin, hematocrit, neutrophil counts, eosinophil counts, SpMet, and pulse rate) showed a significant change between the first and subsequent relapses, but most of these were not showing consistent trends (i.e., increase at one relapse, decrease at the other) and could reflect stochastic variations not related to relapses (Supp. Tables 5 & 6). However, red blood cell counts, hemoglobin, and hematocrit showed consistent trends with increasing values upon each relapse rank, potentially reflecting recovery from the initial acute infection at enrollment over the 90 days (Supp. Fig. 3 & Supp. Table 7). Importantly, inter-individual variability had a substantial impact across nearly all clinical measures, underscoring the heterogeneity of host responses to repeated *P. vivax* infections (Supp. Table 8).

### Genome sequencing confirms that many recurring parasites were not present in the initial infection and highlights the complex relationships among parasites

**Fig 2.**
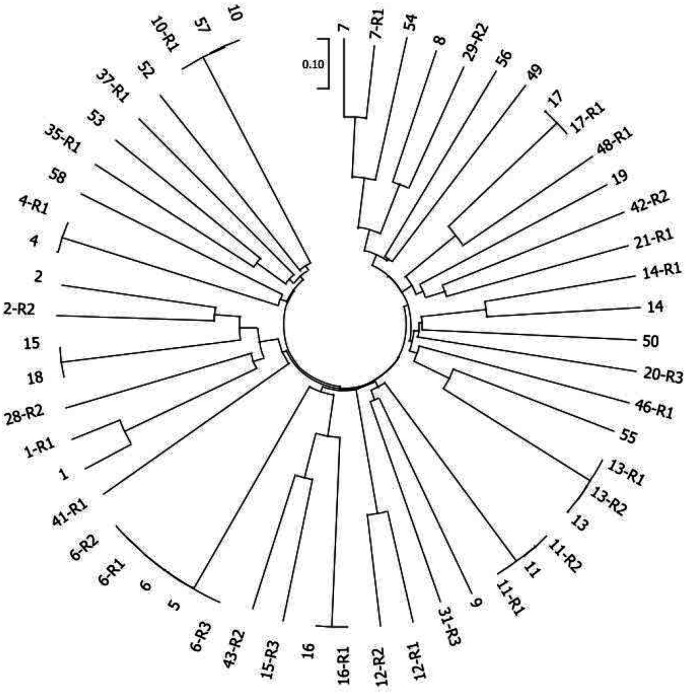
Phylogenetic relationships among *Plasmodium vivax* isolates collected at enrollment and across relapses. Neighbor-joining tree constructed using the fraction of the genome shared IBD between each pair of parasites (n = 56 *P. vivax* monoclonal infection). R1, R2, and R3 denote the first, second, and third relapse episodes, respectively.

We sequenced the parasites’ genomes from all blood-stage infections (i.e., at enrollment and relapses) and obtained high sequence coverage for 99 infections (> 50X for 47 initial infections and 52 relapses), which were then further analyzed. Surprisingly, we observed the same parasite genotype present in different individuals: 3 distinct genotypes were each observed in two different patients at enrollment (i.e. patients 15 and 18, Fig. 2). This pattern, which contrasted sharply with the high genetic diversity reported in previous genomic studies in Cambodia^15^, could indicate a recent reduction in genetic diversity of the Cambodian *P. vivax* population and is consistent with the dramatic reduction in *P. vivax* prevalence in Cambodia in recent years^1^. Note that the reduced diversity observed might have been exacerbated by the recruitment strategy and relocation requirement that could have increased the probability of participants being recruited from the same social circles and/or being infected at the same time.

This apparent reduction in genetic diversity was also evident in the overall decrease in polyclonal infections. Using allelic variations observed within infections, we determined the clonality of each infection. Out of the 47 initial infections that could be rigorously analyzed, 26 were deemed monoclonal (55%), while 21 (45%) were deemed polyclonal, whereas previous studies showed a higher proportion of polyclonal infections^15^. Interestingly, among 52 relapse infections analyzed (24 R1s, 16 R2s, and 12 R3s, denoting first, second, and third relapses, respectively), 22 (42%) were also polyclonal, a proportion similar to that observed at enrollment (Fisher’s exact test, p = 0.84). This observation, which is consistent with our previous study^15^, may indicate that multiple hypnozoites in one individual have reactivated at the same time (or shortly one after the other), and emphasizes the high burden of hypnozoites in these patients.

We next analyzed genetic relationships among parasites by estimating IBD using 56 monoclonal infections for which the parasite genotypes could be unambiguously determined. In 6 patients with a monoclonal initial infection and at least one monoclonal relapse, the recurring clone differed from the clone present at enrollment, confirming that the parasite recurrences were not due to recrudescence of resistant parasites (but see also the discussion below). Overall, we observed all possible relationships of relapsing parasites: some of the parasites were identical to those present in the initial infection; in three cases, the relapsing parasites had genetic relatedness indicative of having a sibling or parent-offspring relationship with the initial infection (indicating that they likely derived from the same mosquito bite); and in three cases the relapsing parasites were unrelated to the initial infection (Fig. 2).

### Polyclonal infections are associated with an increased risk of relapses

**Fig. 3.**
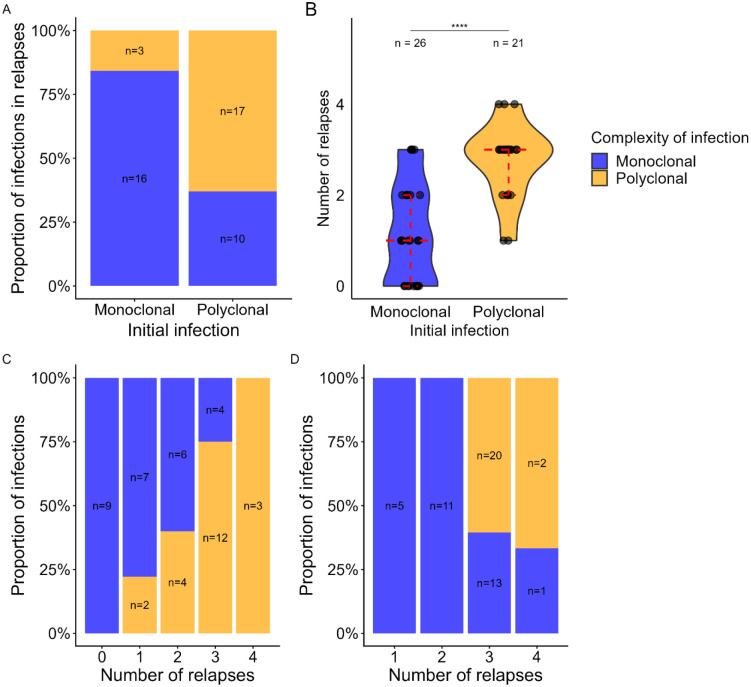
Association between initial infection clonality and frequency of relapses in *P. vivax*. **A**. Clonality of relapses based on initial infection. The x-axis shows whether the initial *P. vivax* infection is monoclonal or polyclonal, while the y-axis represents the clonality of the relapsing infections. Blue indicates monoclonal infections, and orange indicates polyclonal infections. **B**. Comparison of the total number of relapses observed during the study period according to clonality of the initial infection. Individuals with a polyclonal initial infection experienced a significantly higher number of relapses than those with a monoclonal infection (Mann–Whitney, *p* < 0.0001). **C**. Clonality of the initial infection at enrollment stratified by the number of relapses detected during follow-up. The proportion of monoclonal infections at enrollment decreased with increasing number of relapses (Fisher’s exact test, *p* = 0.0003). **D**. Clonality of relapsing infections according to the number of relapses experienced during follow-up. The proportion of monoclonal infection in relapses significantly decreased (Fisher’s exact test, *p* < 0.0001) as the number of relapses increased.

Interestingly, the proportion of monoclonal relapses was higher in patients with a monoclonal initial infection (84%, 16/19) compared to those with a polyclonal initial infection (Fisher’s exact test, 37%, 10/27, p = 0.002) (Fig. 3A). In addition, patients with monoclonal infection at enrollment had fewer relapses detected during follow-up (median 1, IQR: 0–2) compared to patients with a polyclonal initial infection (median 3, IQR: 2–3, p < 0.0001) (Fig. 3B). The proportion of monoclonal infections at enrollment was also significantly different between patients with different number of relapses during the follow-up (Fisher’s exact test, p=0.0003) and decreased with increasing number of relapses: 100% of patients with no relapses had a monoclonal infection at enrollment (9/9), compared to 78% of patients with 1 relapse (7/9), 60% of patients with 2 relapses (6/10), 25% of patients with 3 relapses (4/16) and 0% of patients with 4 relapses (0/3) (Fig. 3C).

Similarly, the proportion of monoclonal relapses differed between patients with varying number of relapses detected during the follow-up (Fisher’s exact test, p <0.0001) and decreased with increasing number of relapses: 100% (5/5) of relapses in patients with 1 relapse, 100% in patients with 2 relapses (11/11), 39% in patients with 3 relapses (13/33) and 33% in patients with 4 relapses (1/3) (Fig. 3D).

### Parasite growth rates during relapses vary between individuals and differ according to parasite-relatedness

For each relapse, we estimated the parasite growth rate as the slope of the increase in parasitemia from the initial qPCR-positive detection of a relapse to the point at which parasites became microscopically detectable, and patients received another artesunate course. In all relapses analyzed in this study, parasitemia consistently increased until becoming microscopically detectable, and no relapse remained sub-microscopic. Parasite growth rates, however, varied widely across relapse episodes, with slopes ranging from 0.04 to 0.89.

**Fig. 4.**
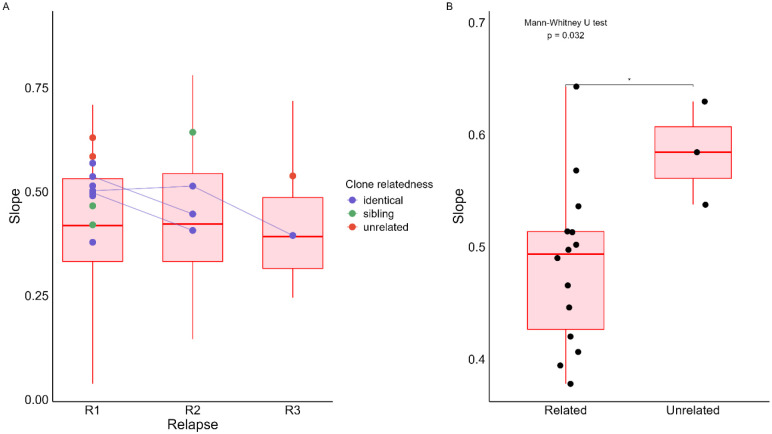
Relationship between parasite growth rate upon relapses and parasite relatedness in *P. vivax*. **A**. Comparison of the growth rate of parasites (y-axis, determined as the slope of the increase in parasitemia over time) across sequential relapses. No significant difference in slope was observed across relapse ranks (linear mixed regression, p=0.23). Colors highlight only the relatedness of clones compared to the initial infection, and the connected dots represent relapses from a given patient. **B**. Comparison of the slope in monoclonal infections between genetically related and unrelated clones compared to the initial infection. The slopes differed significantly between related and unrelated clones (Mann-Whitney, *p* = 0.032). In both panels, box plots represent median (and IQR) slopes.

To evaluate potential determinants of parasite growth rate, we applied a linear mixed-effects model with relapse episodes as a fixed effect and patient ID as a random intercept. The analysis showed no significant difference in parasite growth rates across relapse episodes (F(2, 55.14) = 1.52, p = 0.23; Fig. 4A).

Then, we assessed whether host clinical parameters were associated with parasite growth rate, including hematologic variables (hemoglobin levels) and leukocyte populations (neutrophil, eosinophil, basophil, lymphocyte, monocyte, and platelet counts). None of these variables was significantly associated with parasite growth rate (Supp. Table 9). However, the random effect of patient ID explained a substantial proportion of the variability in slopes, indicating that parasite growth dynamics differed markedly between individuals.

We then evaluated whether parasite characteristics could be associated with parasite growth rates. First, we assessed the impact of infection clonality. Considering all relapse episodes across all patients, the mean slope of monoclonal relapses (0.48 ± 0.1) was not different from the mean slope of polyclonal relapses (t-test, 0.43 ± 0.13, p=0.145). Similar results were obtained when analyses were restricted to 1^st^, 2^nd^, or 3^rd^ relapse (Supp. Fig. 4).

Finally, we tested whether the genetic relatedness between relapsing parasites and those present at enrollment influenced parasite growth rates in monoclonal infections. Interestingly, despite the small sample size, parasite growth rate was associated with the relatedness to the initial infections: relapses caused by genetically unrelated parasites exhibited significantly faster growth rates than those caused by related parasites (Mann-Whitney, p=0.032) (Fig. 4B). This difference remained significant when the analysis was restricted to the first relapse, both when identical and sibling parasites were grouped together (p = 0.036, Supp. Fig. 5A), and when identical and sibling parasites were analyzed separately (Kruskal-Wallis, p = 0.023) (Supp. Fig. 5B). Note however that the sample size is quite small and these results should be verified in other studies to be validated.

### Low-density relapse infections can efficiently transmit to mosquitoes

We performed membrane feeding of *A. dirus* mosquitoes with blood from 40 patients at enrollment and from 36 relapses (occurring in 24 patients). Blood was collected within 24 to 48h after relapses were confirmed by microscopy and prior to artesunate re-treatment. Patients’ sera were replaced with malaria-naïve sera to evaluate the intrinsic transmissibility of parasites during relapses. Additionally, in 18 relapses (occurring in 12 patients), feedings were performed directly from patients’ blood (without serum replacement) (Supp. Fig. 6).

Overall, relapsing parasites were successfully transmitted to mosquitoes, defined by the detection of at least one oocyst at day 6 post-feeding. Without serum replacement, transmission rates were 36% (4/11) at enrollment, 25% (2/8) at R1, 0% (0/6) at R2, and 25% (1/4) at R3. With serum replacement, transmission rates increased to 80% (32/40) for enrollment, 47% (8/17) for R1, 67% (8/12) for R2, and 57% (4/7) for R3 (Supp. Fig. 6 & 7). The lowest parasite density associated with successful transmission with serum replacement was 96 parasites/μl (as measured by thick film), resulting in 1 infected mosquito with 1 oocyst out of 20 examined (Supp. Table 10). These findings show that, even at low parasitemia and shortly upon a relapse, *P. vivax* can be successfully transmitted to mosquitoes.

**Fig. 5.**
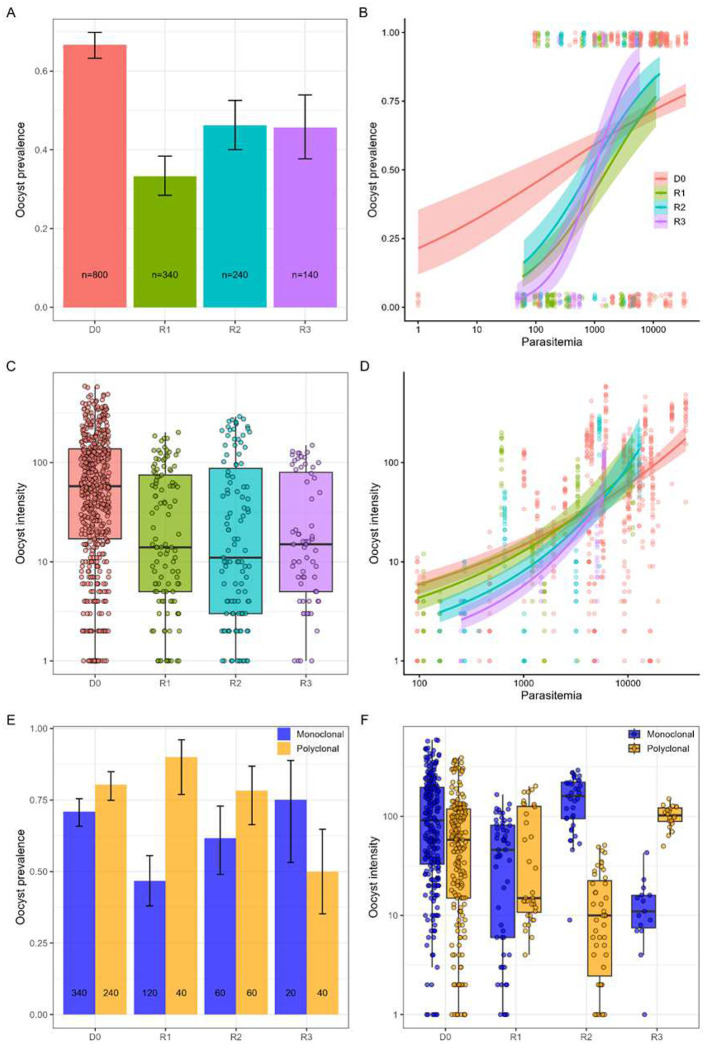
The effect of relapse episode, parasite density, and complexity of infection on *P. vivax* transmission to *Anopheles dirus* in membrane-feeding assays. **(A-B)** Oocyst prevalence and **(C-D)** oocyst intensity measured at 6 days post-blood meal (dpbm). **(A, C)** Distribution of oocyst prevalence and intensity across infections (D0, at enrollment; R1, first relapse; R2, second relapse; R3, third relapse). **(B, D)** Relationship between parasitemia and oocyst prevalence or intensity across infection episodes. Each point denotes an individual mosquito. Lines represent model-fitted estimates (logistic regression for prevalence; quasi-Poisson for intensity) with 95% confidence intervals. **(E-F)** Oocyst prevalence **(E)** and oocyst intensity **(F)** at 6 dpbm stratified by complexity of infection (monoclonal vs. polyclonal) across infection episodes. Error bars represent 95% confidence intervals of the proportion in oocyst prevalence.

To further explore factors influencing mosquito transmission, we analyzed the effects of parasitemia, infection episodes (including initial infections and relapses), age, and sex of patients on oocyst prevalence and intensity. Neither age (LRT $\chi_{1}^{2}=0.09$, p =0.76) nor sex (LRT $\chi_{1}^{2}=2.67$, p = 0.10) had a significant effect on oocyst prevalence, as well as on oocyst intensity (age: LRT $\chi_{1}^{2}=0.67$, p=0.41; sex: LRT $\chi_{1}^{2}=0.74$, p = 0.39). In contrast, the infection episodes showed a significant influence on oocyst prevalence (LRT $\chi_{1}^{2}=10.94$, p < 0.001, Fig. 5A & 5B). Mosquito infection probability also increased strongly with parasitemia (LRT $\chi_{1}^{2}=67.7$, p < 0.001, Fig. 5B). In addition, there was a significant interaction between the infection episodes and parasitemia (LRT $\chi_{1}^{2}=96.6$, p < 0.001, Fig. 5B). Specifically, both at enrollment and upon relapses, the associated differences in oocyst prevalence depended on parasite density: at low parasitemia, R1 and R2 resulted in more infected mosquitoes; however, at high parasitemia, R3 produced the most infected mosquitoes (Fig. 5B).

Oocyst intensity was associated with infection episodes (LRT $\chi_{3}^{2}=60.8$, p < 0.001, Fig. 5C & 5D), parasitemia (LRT $\chi_{1}^{2}=477.8$, p < 0.001, Fig. 5D), and their interaction (LRT $\chi_{3}^{2}=12.8$, p < 0.001, Fig. 5D), indicating infection-specific differences in the relationship between parasitemia and oocyst intensity (Fig. 5D).

We further explored the interactive effect of the complexity of infection (monoclonal vs. polyclonal) and infection episodes on mosquito transmission potential. A significant interaction was observed for both oocyst prevalence (LRT $\chi_{3}^{2}=24.9$, p < 0.001; Fig. 5E) and oocyst intensity (LRT $\chi_{3}^{2}=67.57$, p < 0.001, Fig 5F). Polyclonal infections increased the probability of mosquito infection at D0, R1, and R2, but showed the opposite trend at R3. For oocyst intensity, polyclonal infections yielded fewer oocysts at D0, R1, and R2, potentially reflecting competitive interactions during parasite development, but more oocysts at R3. Caution is warranted for R3 findings due to the limited number of patient samples (n = 3 MFA, 60 dissected mosquitoes). Due to the small sample size, infection episodes were excluded, and we analyzed the effects of parasitemia and the complexity of infection on mosquito infection. Interestingly, there is a significant effect of parasitemia (LRT $\chi_{1}^{2}=20.37$, p < 0.001, Supp. Fig 8A) and non-significant on the complexity of infection (LRT $\chi_{1}^{2}=1.22$, p = 0.27, Supp. Fig 8A), but a significant interaction effect between parasitemia and complexity of infection on oocyst prevalence (LRT $\chi_{1}^{2}=37.3$, p < 0.001, Supp. Fig 8A). However, while parasitemia showed a positive effect on oocyst intensity (LRT $\chi_{1}^{2}=73.1$, p <0.001, Supp. Fig 8B), the complexity of infection (LRT $\chi_{1}^{2}=3.7$, p =0.05, Supp. Fig 8B) showed a marginally significant effect, but not their interaction (LRT $\chi_{1}^{2}=1$, p = 0.31, Sup Fig. 8B). Together, this analysis emphasized that at low parasitemia, the polyclonal infections are associated with high mosquito infection probability but decreased the oocyst intensity at high parasitemia.

## Discussion

Our unique study design enables robust characterization of *P. vivax* relapses in patients living in malaria-endemic areas, excluding reinfections by relocating patients to a transmission-free area and minimizing the masking effect of post-treatment prophylaxis by using a short-lived antimalarial. By combining frequent and sensitive monitoring of parasite recurrences, genomic analyses, and membrane-feeding assays, we document both the high frequency of relapses and their substantial transmission potential. These findings underscore the importance of relapses in sustaining malaria transmission in endemic regions and the challenges facing vivax malaria elimination.

We interpreted the parasite recurrences after supervised artesunate treatment as evidence of relapses derived from the reactivation of dormant hypnozoites rather than recrudescence of resistant or persisted parasites. Relapses appear to be the most parsimonious hypothesis based on (i) the successful clearance of all blood stage parasites within three days of the artesunate treatment initiation, and the similar clearance of all recurrences using the same drug regimen, which supports the absence of artesunate resistance^27^, (ii) patients who received the same artesunate treatment and a high dose of primaquine remained free of parasites for at least 60 days^17^, and (iii) some parasites re-occurring post-treatment were genetically distinct from the parasites initially present. However, it is not possible to entirely rule out complex scenarios whereby primaquine may also have a synergistic or prophylactic effect against blood-stage parasites, and recrudescent parasites were rare in some initial infections and therefore mis-detected by whole-genome sequencing. In addition, while we cannot completely rule out that recurrences are originating from a splenic or bone marrow reservoir of parasites, we believe that the genomic analysis of these recurrences and the known pharmacokinetics and tissue distribution of artesunate^28,29^ are more consistent with relapses from hypnozoite reactivation. Our observation of genetically identical parasites in different patients complicates even further the classification of whether recurrences come from relapses, recrudescence, or reinfections. Probabilistic approaches to classify these recurrences have recently been proposed and may need to be adapted to the epidemiological context of a given area to account for reduced overall diversity^30,31^.

Most patients (81%) had at least one relapse within 90 days, with the majority experiencing multiple relapses. We observed extensive variability in the timing and frequency of relapses which might reflect the heterogeneous hypnozoite burden among individuals (i.e., their cumulative exposure history), possibly compounded by the patient immunity (that may clear some relapsing parasites before their detection) and the parasite genetic diversity (which could contribute to variable periods of dormancy as observed in tropical vs. temperate *P. vivax* isolates). While our findings confirm early studies showing that relapses in tropical *P. vivax* occur frequently and start shortly after the primary infection, they also demonstrate that even under carefully controlled conditions, relapse dynamics vary substantially between individuals^32–35^.

The association between polyclonal infections and increased relapse frequency is consistent with our previous study, which highlighted the complexity of the hypnozoite reservoir^15^. The polyclonal relapses and the heterologous relapses indicate that multiple hypnozoite genotypes were established during prior infections and reactivate independently over time. Overall, our results suggest that the complexity of infection may serve as a proxy for heterogeneity of the hypnozoite reservoir in terms of size (hypnozoites accumulated through multiple past infections) or diversity (some genotypes relapse more than others) and relapse risk^13–15,36^.

Parasite growth rates upon relapses were strongly influenced by individuals but were not significantly impacted by either the relapse ranks or hematologic parameters. In addition, unrelated parasites multiplied significantly faster than parasites genetically related to those present in the initial infection. These results suggest that while there is little evidence of rapid acquisition of immunity against *P. vivax*, strain-specific immunity may (at least partly) control relapsing infections^37^. Note that these results were obtained from a small sample size and should be validated in further studies. This observation is nevertheless consistent with recent results from controlled human malaria infection studies, which show rapid acquisition of clinical immunity without complete suppression of parasite replication^38^.

A key finding of our work is that relapse infections, even at low parasitemia and in the absence of symptoms, can lead to efficient parasite transmission to mosquitoes. When serum was replaced, parasites from the majority of relapse samples were successfully transmitted to vectors. Even without serum replacement, when naturally acquired transmission-blocking immunity is maintained, a substantial number of relapses (17%, 3/18) were infectious. (Supp. Fig. 7). Our analyses indicate that parasitemia (used here as a proxy for gametocytemia) was the main driver of mosquito transmission. Interestingly, at low parasitemia, polyclonal infections were associated with increased mosquito infection probability but decreased oocyst intensity at high parasitemia (Supp. Fig. 8). This suggests, at least at low parasite density, that polyclonal infections may have greater potential for recombination within the mosquito midgut, thereby enhancing the likelihood that some resulting parasite progeny will successfully establish in the mosquito gut. However, competitive interactions between these more diverse parasite genotypes may also occur, limiting oocyst development and resulting in lower oocyst counts. The epidemiological consequences of these findings are unknown and should be explored in larger datasets. Regardless of the impact of multiplicity of infections, our results demonstrate that relapses contribute directly to the infectious reservoir and can sustain transmission, further highlighting the necessity to target hypnozoites vivax malaria elimination.

## Supplementary Material

List of supplementary figures and tables

Supp. Figures

Supp. Fig. 1. Distribution of *P. vivax*-infected patients by number of relapses detected over the 90-day follow-up.

Supp. Fig. 2. Parasitemia comparison between infection episodes (including D0, R1, R2, and R3)

Supp. Fig. 3. Significant overall effect of the relapse ranks on six parameters, including red blood cell counts, hemoglobin, neutrophil counts, eosinophil counts, SpMet, and pulse rate.

Supp. Fig. 4. Comparison of the growth rate (slope) of *P. vivax* between monoclonal and polyclonal samples in all of relapse ranks (R1, R2, and R3).

Supp. Fig. 5. Comparison of growth rates of parasites upon first relapse in monoclonal infections.

Supp. Fig. 6. Effect of the infection episodes (D0, R1, R2, R3) on *P. vivax* transmission to *Anopheles dirus* in membrane-feeding assays without serum replacement.

Supp. Fig. 7. Infectiousness (proportion of MFA resulting in at least one infected mosquito) as a function of infection episodes with serum replacement.

Supp. Fig. 8. Effect of parasitemia and complexity of infection (monoclonal vs. polyclonal) on mosquito infection.

Supp. Tables

Supp. Table 1. Parasite clearance following artesunate treatment at enrollment and upon recurrences. Participants with detectable parasitemia by microscopy are indicated.

Supp. Table 2. Poisson regression model to predict whether clinical parameters at enrollment are associated with the total number of relapses per patient.

Supp. Table 3. Logistic regression model to predict whether clinical parameters at enrollment could impact the risk of having relapses or not.

Supp. Table 4. Proportion of patients with abnormal clinical parameters on the day of enrollment and at each relapse.

Supp. Table 5. Characteristics of clinical parameters of patients at enrollment and at each relapse.

Supp. Table 6: Overall effect of relapse rank on the clinical parameters.

Supp. Table 7. Impact of the relapse rank on clinical parameters at the individual level.

Supp. Table 8. Likelihood ratio test of the random effect of patient ID on clinical and hematological parameters.

Supp. Table 9. Linear mixed-effects regression model with random intercept of patient ID, to examine whether the relapse ranks and clinal parameters could influence the growth rate of parasites (slope).

Supp. Table 10. Summarized results of the membrane feeding assays to evaluate transmission to A. dirus mosquitoes upon infection episodes (D0, R1, R2, R3).

Supplementary Files

This is a list of supplementary files associated with this preprint. Click to download.

- Supplementalinfo06March2026acfinal.docx
